# An online phage therapy bibliography: separating under-indexed wheat from overly indexed chaff

**DOI:** 10.3934/microbiol.2017.3.525

**Published:** 2017-06-27

**Authors:** Diana R. Alves, Stephen T. Abedon

**Affiliations:** 1 School of Pharmacy and Biomolecular Sciences, University of Brighton, Brighton BN2 4GJ, UK; 2 Department of Microbiology, the Ohio State University, 1680 University Dr., Mansfield, OH 44906, USA

One hundred years have passed since the discovery of bacteriophages (phages) by Frederick Twort and Felix d'Hérelle. Over that century an enormous amount of phage research has been undertaken globally—based on the exquisite ability of phages to both infect and kill bacterial cells—in a chase to better understand life generally and also to use phages as tools to eradicate problematic bacteria. The latter typically is described as phage therapy, bacteriophage therapy, or some approximation of phage-mediated biological control of bacteria (here, generally, phage therapy).

After the discovery of penicillin and subsequent dazzle of antibiotic outcomes on bacterial diseases, phage therapy research had seen a deceleration. This decline, however, was predominantly a Western phenomenon while phage therapy research as well as clinical practice continued to take place especially in the former Soviet Union and, subsequently, in Poland [1]. In the last two decades, however, study of phages as antibacterial agents has reemerged more globally as a “novel” but nonetheless quite plausible means of treating bacterial infections, especially where more conventional approaches are not working. Indeed, antibiotic overuse and associated bacterial resistance has coincided with an exponential increase in the number of publications that mention phage therapy [2]. Phage therapy, and related biocontrol of bacteria within other, less clinical contexts [3], nonetheless remains a developing discipline, one in which communication among researchers is of paramount importance as techniques are both developed and refined [4]. Thus, and despite an enormous number of reviews which have come out on phage therapy over the past decade-plus, it is both crucial and increasing difficult to keep on top of this field, an issue which we are in the process of addressing, as outlined here.

In 2010 an editorial was published in Nature Reviews Microbiology titled, “Raiders of the lost articles” [5]. There the author laments the limitations on citation search tools, as well as access to older articles once citations in fact are identified. The field of phage therapy was chosen by way of example. This we can speculate was due to a combination of its long history, current biomedical relevance (i.e., as discussed above), and also because the act of applying phages to bacteria, as a technique, has not become obsolete as new technologies have emerged. Indeed, we can add that the technique in principle requires sufficiently little in the way of equipment that labs which lack the funds to publish in prominent, well-indexed locations, nonetheless can do excellent work. In addition, we can speculate that phage therapy publications are unusual in terms of the breadth of journals in which they can be found. As a consequence, there is a lot of even English-language phage therapy work out there that few seem to know about, despite the existence of sophisticated indexing, including work that is older than any of the researchers practicing in the field [6].

Lack of awareness of what has been published on phage therapy, both past and present, is both unfortunate and potentially enormously wasteful. As noted in [5]:

In many scientific fields there seems to be an increasing lack of appreciation of the early literature around which the field has grown. As a consequence, junior scientists entering a field, who might be unaware of some of the work of the early pioneers, risk wasting a great deal of time and money asking scientific questions that are baseless or have already been answered. …the next generation of scientists must be encouraged to engage more fully with the older literature. Not only will this provide individuals with a greater appreciation for the history of their given field, but it will also help to inform their current work and the scientific questions that they ask. If we fail at this task, then we face the very real prospect that much hard-won knowledge will be lost.

Thus, we face a problem, not limited to but nonetheless highly relevant to phage therapy, one in which, in a sense, a certain degree of ignorance is being institutionalized by limitations in the power of information technology.

Another problem which perhaps is more specific to phage therapy is that of terminology. Phage therapy has been around, as noted, for nearly 100 years, but has not existed during that time as a highly coherent field. Indeed, the simplicity of using phages as antibacterial agents allows entry from many different fields, really anybody with a bacterium which they would like to reduce in numbers. As a consequence, not everyone calls phage therapy “phage therapy”. To a degree, this is very legitimate since the term “therapy” should be limited to describing situations which are in fact therapeutic, such as clinical or veterinary use of phages. Other types of phage therapy should be described as forms of biocontrol or, more formally, as phage-mediated biocontrol of bacteria [3]. In terms of indexing, however, both “phage therapy” and “biocontrol” are problematic since, on the one hand, some prefer “bacteriophage therapy”, and even other terms or phrases (e.g., “control” or “treatment”). On the other hand, biocontrol can also be listed instead as “biological control”, and there is an entire field of biocontrol (or biological control) which has little to do with either phages or bacteria, or indeed sometimes does have something to do with phages, but not always strictly the use of phages as antibacterial agents.

The result is a difficulty in finding phage therapy “wheat”. A “phage therapy” search on PubMed, in quotes, yields 645 references, as this is being written, while a “bacteriophage therapy” search yields 214, “bacteriophage therapy” NOT “phage therapy” yields 141, and “phage therapy” NOT “bacteriophage therapy” yields 572 references. In addition, not all journals are indexed through PubMed, especially papers which lean less towards therapy and more towards biocontrol, and in particular towards applications in aquaculture along with the biological control of foodborne pathogens.

The problem with “chaff” stems in part from the noted issue of generality of the use of “biocontrol” as a term. This is seen in searching Google Scholar, even when limiting searches to those that mention “phage” or “bacteriophage”. The following search, without the external quotes, thus yields over twenty thousand hits: “(phage OR phages OR bacteriophage OR bacteriophages) AND (biocontrol OR “biological control”)”. A second issue is that “phage therapy” is a “hot” buzzword to use in phage publications, particularly when describing phages with potential but not yet demonstrated phage therapy application, where the latter simply are not phage therapy publications. Thus, a Google Scholar search on “phage therapy” OR “bacteriophage therapy” (with the quotes) yields approximately 9,000 hits, which probably is seven or so thousand in excess in terms of actual phage therapy work, or reviews. Given use of alternative descriptive terms, perhaps there are hundreds of references missing as well. The issue, then, is one of identifying phage therapy or phage-mediated biocontrol “wheat” from faux “phage therapy” and “biocontrol” chaff, which, to the extent we so far can tell, is simply a matter of putting in sufficient time and effort. Here then we describe both the culmination of nearly 20 years of tracking down phage therapy references along with plans to keep the process going, on a year-to-year basis, as an online Phage Therapy Bibliography.

We have compiled and have made freely available references to primary research articles, reviews, books, and book chapters covering the subject of phage therapy and, more generally, phage-mediated biocontrol of bacteria. This can be found at publications.phage-therapy.org or phage-therapy.org/literome. (Historical note: To our knowledge, the term “literome”, at least as applied to the bacteriophage literature, was invented by Ryland Young of Texas A&M University sometime during or prior to 2003, with the earliest such use as indexed on Google Scholar dating to 2006.) The bibliography focuses on English-language publications and is listed in descending-year and ascending-author order. At the moment the bibliography covers the years 1917 to 2016 with a goal of expanding the collection both yearly, i.e., as initiated prior to the end of the following year, and on a more ad hoc basis as we become aware of otherwise missed publications. To achieve these goals, as well as towards error checking, we kindly ask individuals with an interest in phage therapy to look into the bibliography and help us towards making it as complete as possible.

In the bibliography, each reference is complemented with the PubMed link to the article, if available. There the user often can access the article abstract, and in many cases view the entire article as well. References are highlighted (“Open Access!”) for those articles which are of obvious open access availability via PubMed. In addition, we are in the process of providing links, via Google Scholar, which can be helpful especially when PubMed indexing is lacking. At the moment the bibliography comprises approximately 1400 phage therapy-related references, which is remarkable when compared to PubMed which for the same 1917–2016 period, when searching on “phage therapy” (in quotes), provides roughly 600 references. Our aim especially is to provide to phage therapy researchers, including, of course, ourselves, a somewhat complete gateway to a majority of the English-language phage therapy scholarly publications.

A fundamental part of doing research, apart from experimentation, is searching and reading the published literature. It is, obviously, truly difficult to overstate the importance of doing this, though at the same time we are personally aware of how difficult a task this actually can be. The older literature especially should not be overlooked simply for reasons of false perceptions of irrelevance or, instead, of inconvenience. Certainly phage therapy researchers with interests in treating specific organisms should not hesitate in reaching as far back as possible in this literature as it pertains, therapeutically using phages, to those specific organisms. For academic and industry researchers therefore it is crucial to be at least aware of and, ideally, also to have access to previous and pioneering research, where often science was done under fewer institutional pressures, less pressing near-term goals, or indeed fewer limitations on clinical research. Such collective awareness is essential towards expanding our knowledge of phages, the dynamics of their interactions with bacterial cells, their interactions with treated environments, and especially their clinical use. Insufficiencies in such awareness can result in a “polluting” of the published literature with what politely may be described as poorly processed information. Researchers, that is, need to be asking the next questions rather than wasting time and money unknowingly repeating or otherwise obscuring what already has been published. Our hope is that this online phage therapy bibliography, in providing better access to the phage therapy literature, will be helpful towards the development and eventual Western re-acceptance of phages as effective antibacterial agents.
